# RNA Editing Analysis Reveals Methyl Jasmonic Acid Regulation of Fucoxanthin and Fatty Acid Metabolism in *Phaeodactylum tricornutum*

**DOI:** 10.3390/md23020066

**Published:** 2025-02-06

**Authors:** Sihui Huang, Hao Liu, Ruihao Xu, Wangchang Li, Han Yang, Xinlei Bao, Yuqing Hang, Yifu Gong, Yuxiang Zhao

**Affiliations:** 1 Key Laboratory of Marine Biotechnology of Zhejiang Province, School of Marine Sciences, Ningbo University, Ningbo 315200, China; faithuang@163.com (S.H.); 17757429150@163.com (H.Y.); 18725017875@163.com (X.B.); 2 Institute of Bioengineering, Biotrans technology Co., Ltd., Shanghai 201500, China; hao_liu@biotranstech.com (H.L.); ruihao_xu@biotranstech.com (R.X.); liwangchang@biotranstech.cn (W.L.); 3 United New Drug Research and Development Center, Biotrans Technology Co., Ltd., Changsha 410000, China; 4 College of Plant Protection, Hunan Agricultural University, Changsha 410000, China

**Keywords:** *Phaeodactylum tricornutum*, fucoxanthin, unsaturated fatty acids, methyl jasmonic acid, RNA editing, *Lhcr10*, *Phatr3_J43665*

## Abstract

*Phaeodactylum tricornutum* is a marine diatom with significant biotechnological potential, particularly in producing high-value bioactive compounds such as fucoxanthin and unsaturated fatty acids, which possess significant pharmaceutical and nutraceutical properties. However, the naturally low yields of these compounds present a major challenge for large-scale production. Methyl jasmonic acid (MeJA), a plant-derived signaling molecule, has been shown to enhance the biosynthesis of these metabolites in *P. tricornutum*. While transcriptional regulation has been extensively studied, the role of post-transcriptional modifications, such as RNA editing, in mediating MeJA-induced metabolic changes remains largely unexplored. RNA editing can alter nucleotide sequences, leading to functional changes in gene expression and protein activity, thus providing a potential regulatory mechanism for enhanced biosynthesis of target metabolites. In this study, we investigated the role of RNA editing in *Phaeodactylum tricornutum* under methyl jasmonic acid (MeJA) treatment, focusing on its impact on the accumulation of bioactive compounds such as fucoxanthin and fatty acids. We conducted a comprehensive comparative analysis of RNA editing events across MeJA-treated and control groups. Our findings reveal that MeJA treatment induces significant variations in RNA editing levels, affecting key metabolic pathways. Notably, two genes, *Lhcr10* (*Phatr3_J16481*) and *Phatr3_J43665*, were identified as potential contributors to increased RNA editing enzyme activity and to energy metabolism and fatty acid biosynthesis under MeJA treatment. These results provide a foundation for the discovery of molecular mechanisms underlying adaptive responses in *P. tricornutum* and highlight RNA editing as a critical regulatory mechanism in MeJA-induced metabolic reprogramming.

## 1. Introduction

Microalgae are a critical component of marine ecosystems and have gained increasing attention for their potential applications in biotechnology [1,2,3]. As primary producers, they play a vital role in global carbon cycling and serve as a sustainable source of bioactive compounds, including raw materials, nutrients, and biofuels [4,5,6]. These attributes make microalgae indispensable for various industries, including pharmaceuticals, nutraceuticals, and renewable energy [7,8].

*Phaeodactylum tricornutum* (*P. tricornutum*), a marine diatom, is widely recognized as a model organism in microalgal research due to its unique physiological, biochemical, and molecular characteristics [9]. As a pennate diatom, it possesses the ability to accumulate a range of high-value metabolites, such as carotenoids and unsaturated fatty acids [10]. These bioactive compounds not only contribute to its ecological resilience but also hold immense potential for human health applications [11]. The availability of a fully sequenced genome, along with advanced genetic engineering tools, makes *P. tricornutum* an ideal system for studying metabolic regulation and stress responses [12,13]. Unlike many other algal species, *P. tricornutum* demonstrates exceptional plasticity in its physiological and metabolic adaptations to environmental changes, providing researchers with valuable insights into algal biology and potential biotechnological applications [14]. These features have established *P. tricornutum* as a pivotal organism for exploring novel molecular mechanisms, such as RNA editing, which remains underexplored in microalgae.

Fucoxanthin, a carotenoid abundantly produced by marine diatoms, plays an important role in light harvesting during photosynthesis [15]. Beyond its ecological functions, fucoxanthin exhibits a wide array of pharmacological properties, including anti-inflammatory [16], anti-cancer [17], anti-obesity [18], and antioxidant activities [19]. These properties have positioned fucoxanthin as a promising candidate for drug development. Similarly, the unsaturated fatty acids synthesized by *P. tricornutum* are essential for cellular functions and provide significant health benefits, such as improving cardiovascular health [20] and reducing inflammation [21]. Enhancing the yield of these compounds is a key goal in *P. tricornutum* research.

However, the low natural yield and high extraction costs of metabolites like fucoxanthin and unsaturated fatty acids significantly limit their commercial applications. For instance, fucoxanthin is typically present in trace amounts in microalgae, often accounting for less than 1% of dry biomass weight, making large-scale production economically unfeasible [22,23]. The extraction process, which relies on organic solvents and labor-intensive purification steps, further escalates the production costs and limits scalability [24]. Additionally, the heterogeneity of microalgal cultures and sensitivity to environmental conditions introduce variability in fucoxanthin yields, complicating standardization for industrial applications [25]. Recent efforts to optimize cultivation strategies, such as light and nutrient modulation, have shown potential to improve yields but are still far from meeting industrial demand [26,27]. These challenges underscore the urgent need for novel strategies to enhance the accumulation of high-value metabolites in microalgae and improve their extraction efficiency.

Methyl jasmonate (MeJA), a signaling molecule in plants, has been widely studied for its ability to induce the production of secondary metabolites under stress conditions [28,29,30]. In microalgae, MeJA has been shown to enhance the synthesis of high-value compounds, such as astaxanthin in *Haematococcus pluvialis* [31]. Our previous study demonstrated that MeJA treatment has been linked to increased production of fucoxanthin in *P. tricornutum*. Specifically, we determined that an optimal MeJA concentration of 200 μmol/L significantly enhances fucoxanthin yield [32]. However, these studies primarily examined the effects of MeJA on gene expression changes, and the potential role of post-transcriptional modifications, such as RNA editing, remains largely unexplored.

RNA editing is a post-transcriptional process that alters RNA sequences, thereby modulating gene expression and protein functionality [33,34]. It has the potential to significantly impact metabolic reprogramming [35]. By altering RNA sequences, it can lead to the production of protein isoforms with different functionalities, which may enhance or inhibit specific metabolic pathways [36]. This capability offers a promising avenue for improving the metabolic efficiency of microalgae, such as *P. tricornutum*, and could be harnessed to increase the production of valuable bioactive compounds. This phenomenon is widespread across plants [37], animals [38], and microorganisms [39] and plays an essential role in environmental adaptation [40], stress response [41], and metabolic regulation [42]. Despite its importance, RNA editing in microalgae is understudied, particularly regarding its impact on metabolic pathways. Advanced RNA editing sequencing (RE-seq) technologies have now enabled precise detection of RNA editing events, overcoming the limitations of traditional RNA-seq methods.

**Figure 1:**
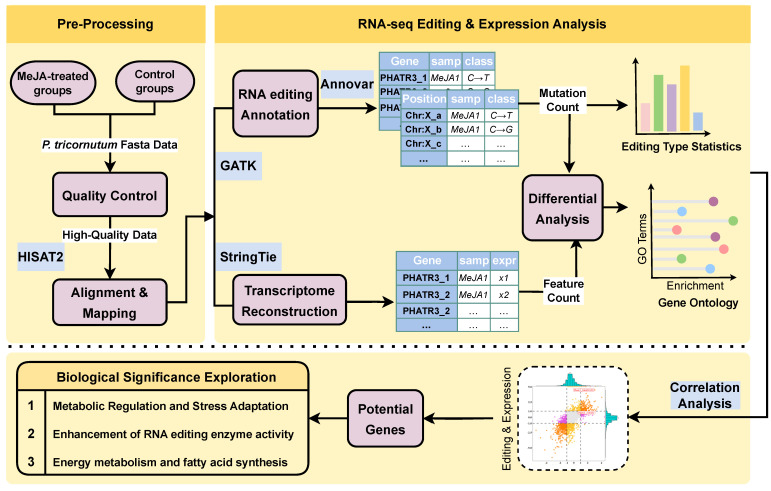
A summary of the workflow used for discovering MeJA-induced genes related to fucoxanthin and fatty acid synthesis using RNA editing analysis.

In this report, we aimed to elucidate the regulatory role of RNA editing in *P. tricornutum* under MeJA treatment, focusing on its impact on metabolite production. To achieve this goal, we conducted a comprehensive transcriptomic analysis of *P. tricornutum* samples from a group treated with 200 μmol/L MeJA and an untreated control group. This study pre-processed the high-throughput transcriptomic sequencing data of MeJA-treated and control samples; identified both RNA editing sites and gene expression levels; and then performed differential RNA editing (DRE) analysis, differential gene expression analysis, Gene Ontology (GO) enrichment analysis, Kyoto Encyclopedia of Genes and Genomes (KEGG) enrichment analysis, and correlation analysis between RNA editing sites and gene expression levels to find candidate MeJA-induced genes related to fucoxanthin and fatty acid synthesis. A summary of this pipeline is shown in Figure 1. This study provides novel insights into the role of RNA editing in regulating metabolic pathways in *P. tricornutum*. It underscores the potential of leveraging RNA editing to optimize the production of high-value metabolites in marine algae, paving the way for future research into RNA editing modulators and their biotechnological applications.

## 2. Results

### 2.1. General Characteristics of RNA Editing Events in Control and MeJA-Treated P. tricornutum Samples

In our previous study [32], we explored the effects of methyl jasmonate (MeJA) on growth and fucoxanthin content in *Phaeodactylum tricornutum*. The results indicated that MeJA treatment, particularly at a concentration of 200 μmol/L, led to a significant increase in fucoxanthin content compared to the control group.

The first step to discover RNA editing mechanism-related candidate genes associated with MeJA-induced metabolic changes in *P. tricornutum* was to obtain high-throughput transcriptome profiles. The RNA-seq raw fasta data of MeJA-treated and untreated *P. tricornutum* samples were obtained from our previous study. All raw sequencing reads from both MeJA-treated and untreated control samples of *P. tricornutum* were subjected to quality control to remove low-quality bases and contaminants. Then, sequencing reads were aligned and mapped to the reference genome using HISAT2. RNA editing sites were identified using GATK and annotated with ANNOVAR. In parallel, transcriptome reconstruction was performed using StringTie to compile and quantify gene expression levels. Both RNA editing and gene expression data are used in differential analyses to detect significant differences between treated and control samples. GO enrichment is then applied to identify affected biological processes. Finally, editing and expression data are integrated for correlation analysis to uncover key candidate genes involved in metabolic regulation.

**Figure 2:**
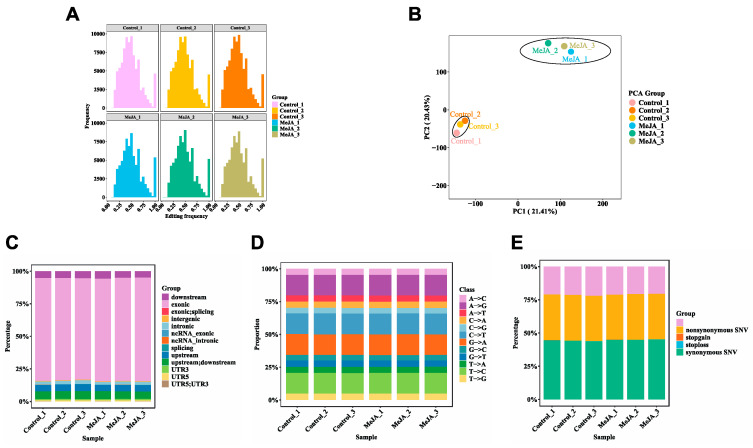
Overall characteristics of RNA editing events. (**A**) Distribution of RNA editing frequencies across control samples and MeJA-treated samples. The *x*-axis represents RNA editing frequency bins, while the *y*-axis indicates the number of RNA editing events in each frequency bin. (**B**) Principal component analysis (PCA) of RNA editing frequencies. Each point in the PCA plot represents a biological replicate. MeJA-treated samples are shown in a blue to green color gradient, and control samples are presented in a red to orange color gradient. (**C**) Genomic region distribution of editing events. Different colors represent various genomic locations, including exonic, intronic, upstream, downstream, and other regions. (**D**) Classification of RNA editing event types. Stacked bars show the proportions of different editing types (e.g., A→C, A→G) in each sample. (**E**) Classification of single nucleotide variants (SNVs) resulting from RNA editing events. Stacked bar plots illustrate variant types, including synonymous SNVs, nonsynonymous SNVs, stop-gain, and stop-loss events.

To investigate the distribution and characteristics of RNA editing events under MeJA treatment, all detected RNA editing sites were systematically annotated and classified (Figure 2). A total of 231,482 editing sites were identified in the MeJA-treated group, compared to 262,911 sites in the control group (Table 1). The editing frequency distribution was analyzed across all samples. Histograms reveal a broad range of editing frequencies in both the control and MeJA-treated groups, indicating baseline RNA editing activity in *P. tricornutum* (Figure 2A). Principal component analysis (PCA) of RNA editing frequencies shows distinct clustering of MeJA-treated samples and control samples, highlighting a clear separation along the PC1 (21.41%) and PC2 (20.43%) axes. This indicates that MeJA treatment induces significant global changes in RNA editing patterns (Figure 2B).

The characteristics of identified RNA editing sites were summarized. The majority of RNA editing events were located in exonic regions, underscoring their potential impact on protein-coding genes. Other notable proportions were found in upstream, downstream, splicing, and ncRNA intronic regions, suggesting that RNA editing may influence gene regulation and function at multiple genomic levels (Figure 2C). RNA editing types were categorized across all samples. C-to-U (C→T or G→A) and A-to-I (A→G or T→A) editing events were predominant, collectively accounting for approximately 80% of all detected editing events. Other editing types, such as A-to-C and A-to-T, were less frequent, reflecting the diversity of the RNA editing landscape in *P. tricornutum* (Figure 2D). Analysis of RNA editing-induced single nucleotide variants (SNVs) revealed that synonymous SNVs were the most prevalent, followed by nonsynonymous SNVs, which can directly affect protein structure and function. Stop-gain and stop-loss variants, although rare, have profound effects by introducing or removing stop codons, potentially altering protein length and functionality (Figure 2E).

### 2.2. Differential Analysis of RNA Editing and Gene Expression

To identify RNA editing differences between MeJA-treated and control samples, we conducted differential analysis at both the RNA editing site level and the RNA editing gene level and compared these results with traditional gene expression differential analysis.

**Figure 3:**
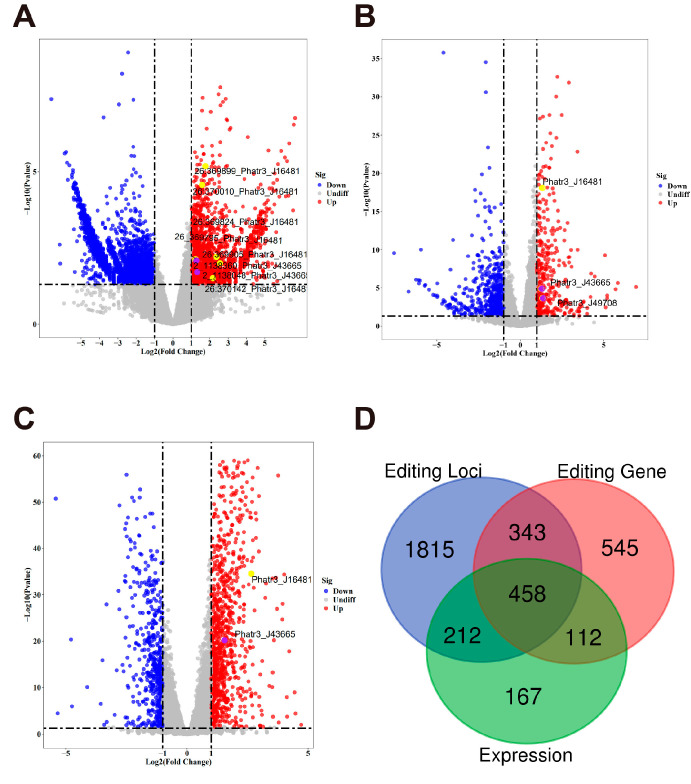
Differential analysis for both RNA editing and gene expression. (**A**–**C**) Volcano plots indicating differential analysis results at different levels: (**A**) RNA editing site level, (**B**) RNA editing gene level, and (**C**) gene expression level. The *x*-axis indicates log2 fold changes, and the *y*-axis indicates statistical significance (-log_10_ *p*-value). Each dot represents a gene or locus: red indicates significant upregulation, blue indicates significant downregulation, and gray represents no significant change. Labeled yellow and purple dots highlight two selected genes of interest, separately. (**D**) Venn diagram illustrating the intersection of differentially RNA editing sites (editing loci), RNA editing-targeted genes (editing gene), and expressed genes (expression).

A total of 5415 RNA editing sites were found to exhibit significant differences (adjusted *p*-value < 0.05) between the MeJA-treated and control groups. These sites were derived from 2827 genes, with 1922 upregulated editing sites from 833 genes, and 3493 downregulated editing sites from 1888 genes. Among them, 106 genes contained both upregulated and downregulated editing sites. This highlights a complex regulatory landscape where RNA editing events within the same gene can exhibit divergent responses to MeJA treatment (Figure 3A). At the gene level, 948 genes were identified as significantly differentially edited, with 346 upregulated genes and 602 downregulated genes (Figure 3B). The number of differentially edited genes is notably lower than the total number of genes contributing to site-level editing events, reflecting the aggregation of multiple editing sites into gene-level summaries. The identified RNA editing based sites and genes, particularly those highlighted in the volcano plots, warrant further investigation to understand their roles in the biological response to MeJA and their potential involvement in metabolic regulation and stress adaptation.

Traditional gene expression analysis identified 1457 differentially expressed genes, with 958 upregulated genes and 499 downregulated genes (Figure 3C). The overlaps and unique components of the three differential analyses were assessed using a Venn diagram (Figure 3D). A total of 457 genes were common across the three analyses, indicating that these genes were regulated at both the RNA editing and transcriptional levels. Among the 948 differentially edited genes, 212 were shared with the 5415 differentially edited sites, while 167 editing genes were unique to gene-level analysis. Conversely, 1815 editing sites were unique to site-level analysis, emphasizing the finer resolution provided by site-level editing data. In total, 112 genes were shared between differentially expressed genes and differentially edited genes, while 343 genes overlapped between differentially expressed genes and site-level editing. In addition, 545 differentially expressed genes were unique to transcriptional regulation, highlighting distinct layers of control by gene expression and RNA editing. These findings demonstrate the complementary roles of RNA editing and gene expression in shaping transcriptomic responses to MeJA treatment. While gene expression provides a broader regulatory framework, RNA editing introduces an additional layer of post-transcriptional control, with site-specific events playing a crucial role in fine-tuning gene function. The substantial number of unique editing sites suggests that RNA editing contributes independently to the regulatory landscape.

### 2.3. Functional Enrichment Analysis of Differential Gene Sets

**Figure 4:**
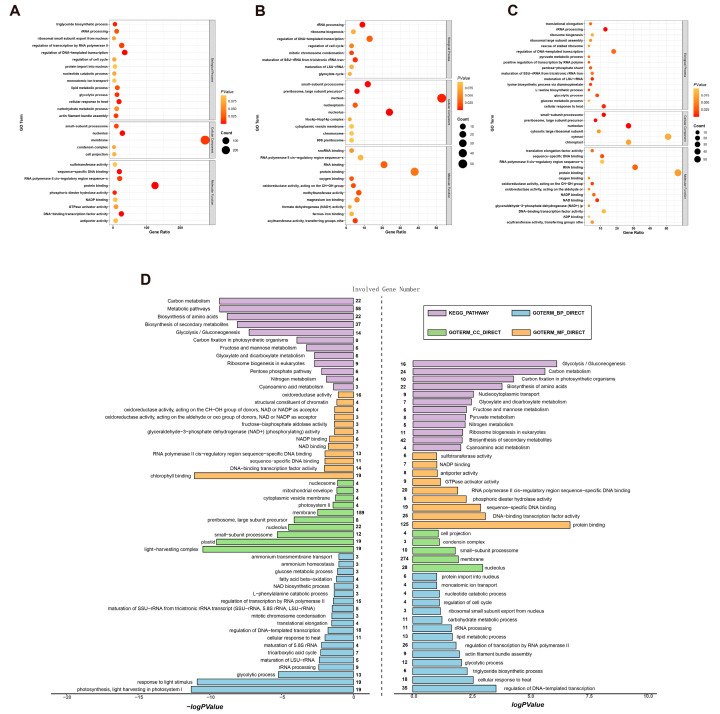
Functional enrichment analysis. (**A**–**C**) GO enrichment analyses for the three differential gene sets: (**A**) RNA editing at the site level, (**B**) RNA editing at the gene level, and (**C**) gene expression. Each bubble chart displays enriched GO terms grouped into three categories: biological processes (BP), cellular components (CC), and molecular functions (MF). Bubble size reflects the number of genes annotated to each term, while the color gradient represents the significance level (*p*-value). These analyses reveal distinct biological processes and pathways impacted by gene expression changes and RNA editing. (**D**) Integrated GO and KEGG enrichment analysis comparing RNA editing (right) and gene expression (left). GO terms are divided into three categories: biological processes (blue), cellular components (green), and molecular functions (orange), while KEGG pathways are shown in purple. The size of each node indicates the number of genes involved, and numbers on the chart represent the total genes associated with each item. This comparative analysis highlights unique and overlapping pathways influenced by transcriptional and post-transcriptional regulation.

To elucidate the biological implications of RNA editing and gene expression changes under MeJA treatment, Gene Ontology (GO) and Kyoto Encyclopedia of Genes and Genomes (KEGG) enrichment analyses were performed for the three differential gene sets: differentially edited genes at the site level, differentially edited genes at the gene level, and differentially expressed genes. These analyses revealed distinct functional categories and metabolic pathways associated with each group (Figure 4).

At the site level, differentially edited genes were enriched in terms related to lipid metabolism, including triacylglycerol biosynthesis (GO:0019432) and lipid metabolic processes (GO:0006629). These findings highlight the role of site-specific RNA editing in fine-tuning lipid metabolic pathways under stress conditions (Figure 4A). Gene-level RNA editing analysis showed no specific enrichment in fatty acid or unsaturated fatty acid pathways. However, broader terms related to gene regulation and cellular processes were observed, suggesting that aggregated RNA editing at the gene level may have a more general regulatory role (Figure 4B). Genes with differential expression also show a broader association with metabolic processes, stress responses, and cellular homeostasis (Figure 4C). The genes were significantly enriched in (GO:0006635), indicating an upregulation of lipid catabolic processes in response to MeJA treatment.

A comparative view of GO and KEGG pathway enrichments for RNA editing site level and gene expression is shown in Figure 4D. RNA editing genes at the site level showed enrichments in pathways related to lipid metabolism, reflecting their involvement in lipid synthesis and modification. For differentially expressed genes, enriched KEGG pathways included metabolic processes, such as fatty acid β-oxidation, reinforcing their role in lipid metabolism. The distinct patterns observed in the GO and KEGG enrichment analyses provide a comprehensive view of the potential impacts of RNA editing on the biological processes in *P. tricornutum*.

### 2.4. Integrated Analysis of RNA Editing and RNA Expression

**Figure 5:**
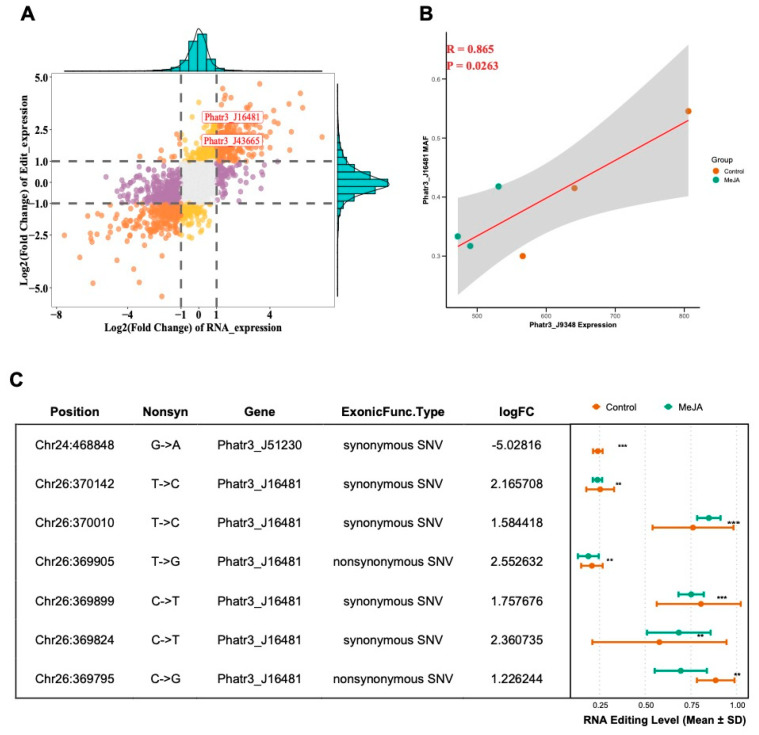
Integrated comparison of RNA editing events and RNA expression. (**A**) Nine-quadrant scatterplot correlating RNA expression changes (*x*-axis) with RNA editing changes (*y*-axis) for individual genes. Each dot represents a gene, with colors distinguishing different correlation statuses. Orange represents genes with log2 fold change significant in both RNA editing and RNA expression. Purple represents genes with log2 fold change significant only in RNA editing but not in RNA expression. Yellow represents genes with log2 fold change significant only in RNA expression but not in RNA editing. Grey represents genes with no significant changes in either RNA editing or RNA expression. Histogram at the top and right edge shows the genes both upregulated in RNA editing and RNA expression. Histogram at the top and left edge shows the genes upregulated in RNA editing and downregulated in RNA expression. Histogram at the down and right edge shows the genes downregulated in RNA editing and upregulated in RNA expression. Histogram at the down and left edge shows the genes both downregulated in RNA editing and RNA expression. Genes of interest, such as those involved in fucoxanthin and fatty acid metabolism pathways, are highlighted. (**B**) Linear regression plot showing the correlation between the gene expression of *Phatr3_J9348* and the mean allele frequency (MAF) of RNA editing sites in *Phatr3_J16481* (*Lhcr10*). The red line represents the best-fit regression, with the shaded area indicating the 95% confidence interval. Correlation coefficient (R) and *p*-value indicate statistical significance. (**C**) Summary of selected single nucleotide variants (SNVs) identified in RNA editing analysis. The table lists gene, chromosome position, nucleotide changes (e.g., synonymous or nonsynonymous SNVs), and statistical significance (*p*-values for both gene and locus levels). Associated dot plots compare RNA editing frequencies (±SD) between the control and MeJA-treated groups, with asterisks marking significant differences (** *p* < 0.01, *** *p* < 0.001). Notable SNVs in *Phatr3_J43665* are highlighted, including one synonymous and one nonsynonymous SNV, providing potential insights into lipid metabolism regulation.

To comprehensively analyze the relationship between RNA editing and RNA expression, a nine-quadrant scatterplot was generated to integrate both data types (Figure 5A). The *x*-axis represents changes in RNA expression (log_2_ fold change), while the *y*-axis denotes changes in RNA editing (log_2_ fold change of mean editing levels). Genes were distributed into nine regions based on their expression and editing patterns, highlighting genes with consistent upregulation or downregulation in both metrics. Among these, genes involved in fucoxanthin metabolism (e.g., *Lhcr10/Phatr3_J16481*) and fatty acid metabolism (e.g., *Phatr3_J43665*) were prominently located in the top-right quadrant, indicating coordinated upregulation in both RNA expression and RNA editing levels.

To further investigate the regulatory mechanisms, a linear regression analysis was conducted between RNA editing levels of key genes and RNA expression of relevant RNA editing enzymes (Figure 5B). Notably, a strong positive correlation was observed between the RNA expression of *Phatr3_J9348* (APOBEC RNA editing enzyme) and the mean allele frequency (MAF) of editing sites in *Lhcr10* (*Phatr3_J16481*) (R = 0.865, *p* < 0.01). This finding provides evidence that the APOBEC RNA editing enzyme, upregulated under MeJA treatment, may mediate the precise editing of photosynthesis-related genes to enhance fucoxanthin synthesis.

Detailed analysis of RNA editing loci revealed significant differences between control and MeJA-treated samples, particularly in fucoxanthin biosynthesis-related genes *Lhcr11* (*Phatr3_J51230*) and *Lhcr10* (*Phatr3_J16481*) (Figure 5C, Table S1). For example, the RNA editing event of a synonymous SNV (G→A, known as C-to-U editing) in *Lhcr11* (*Phatr3_J51230*) at chromosome 24, position 468848, only occurs in control groups (* *p* < 0.001). Another RNA editing event of a synonymous SNV (T→C, known as A-to-I editing) in *Lhcr10* (*Phatr3_J16481*) at chromosome 26, position 370010, exhibited significantly higher MAF in MeJA-treated groups than in control groups (* *p* < 0.001). These editing events likely influence transcript stability or function, contributing to enhanced fucoxanthin production under stress conditions.

Further focusing on fatty acid metabolism-related pathways, differential analysis identified genes such as *Phatr3_J43665* and *Phatr3_J49708* (Table S2), which showed significant differences across RNA expression, gene-level editing, and site-level editing. Among these, *Phatr3_J43665* stands out due to its two RNA editing loci with significantly increased editing frequencies in MeJA-treated samples. These include two rare RNA editing type, a synonymous SNV (G→T) and a nonsynonymous SNV (C→G), both of which may influence transcript structure and stability, thereby enhancing lipid metabolism activity. Table 2 summarizes the detailed characteristics of these RNA editing loci, including chromosomal position, nucleotide changes, and statistical significance.

Together, these results demonstrate that integrating RNA editing and RNA expression analyses enables the identification of critical genes, such as *Lhcr10* and *Phatr3_J43665*, that are co-regulated at multiple molecular levels. These findings highlight the potential of RNA editing as a regulatory mechanism to optimize metabolic pathways, including fucoxanthin biosynthesis and fatty acid metabolism, under MeJA-induced stress conditions.

## 3. Discussion

The choice of *Phaeodactylum tricornutum* as a model organism is grounded in its unique biological and biotechnological relevance [9]. Its well-annotated genome and the availability of molecular tools, such as CRISPR–Cas systems [43], facilitate functional genomic studies. Additionally, *P. tricornutum* thrives under diverse environmental conditions, making it an excellent model for investigating stress-induced metabolic pathways, including the biosynthesis of carotenoids like fucoxanthin. Compared to other microalgae, *P. tricornutum* offers distinct advantages, such as rapid growth, ease of cultivation, and a wealth of genetic information, enabling the integration of multi-omics approaches.

MeJA is a phytohormone that regulates growth and development and plays an important role in the synthesis of relevant compounds under environmental stress. Recent studies have demonstrated its potential to enhance the production of valuable bioactive compounds in medical plants. Mohammad Ali’s team reported that treatment with 1.0 mg/L MeJA, jasmonic acid (JA), as well as gibberellic acid (GA) together increased the accumulation of artemisinin in cell suspension cultures of *Artemisia absinthium L* [44]. Ana-Belén Sabater-Jara and others found that by adding both MeJA and cyclodextrins, the production levels of taxol biosynthesis in *Taxus bacata* were 55 times higher than in non-elicited cultures [45]. Utilizing MeJA to treat Chinese chives (*Allium tuberosum*), Cheng Wang’s team reported that there was a significant increase in total sugars, essential and non-essential amino acids, sulfur-containing amino acids, phenolic content, and carotenoid content, which in turn enhanced the antioxidant activity as well as the nutritional and medicinal value of the plants [46]. The treatment of MeJA also increases the production of the bioactive metabolites in diatoms. For example, Dónal Mc Gee’s team found that 10 μM MeJA treatment enhanced fucoxanthin and lipid production in *Stauroneis sp.* by modulating antioxidant responses, photosynthetic processes, and metabolite fluxes [47]. Our previous study demonstrated that a concentration of 200 µmol/L MeJA was optimal for maximizing fucoxanthin production in *Phaeodactylum tricornutum*, achieving a 139% increase in fucoxanthin content per gram of dry weight of algal cells compared to the control group [32]. This study provides a foundation for our work, as we utilized the same optimal concentration of MeJA to investigate its effects in increasing the production of fucoxanthin from the perspective of RNA editing. By exploring RNA editing events induced under these conditions, we aimed to uncover the post-transcriptional regulatory mechanisms underlying fucoxanthin biosynthesis, offering novel insights into how MeJA modulates metabolite production at the RNA level.

Fucoxanthin, a xanthophyll carotenoid abundant in *P. tricornutum*, holds immense potential for pharmaceutical and nutraceutical applications due to its anti-inflammatory, anti-cancer, and antioxidant properties [48]. Similarly, unsaturated fatty acids synthesized by *P. tricornutum*, such as eicosapentaenoic acid and docosahexaenoic acid, are critical for human health, offering cardiovascular and anti-inflammatory benefits [49]. The commercial significance of these compounds underscores the importance of understanding and enhancing their biosynthetic pathways.

Understanding the role of RNA editing in metabolic reprogramming opens new possibilities for enhancing the production of bioactive compounds in microalgae. By modulating RNA editing events, it may be possible to rewire metabolic pathways to favor the synthesis of desired products. This approach could complement other metabolic engineering strategies and provide a more flexible and precise tool for optimizing microalgal metabolism. Additionally, RNA editing is crucial in environmental adaptation and stress responses. For instance, in wheat, RNA editing has been shown to induce structural and functional changes in the NAD9 protein under drought stress, highlighting its importance in stress adaptation [50]. In microalgae, similar mechanisms may be at play, where RNA editing could modulate the expression and function of proteins involved in stress responses, potentially enhancing their resilience to environmental changes. This could be particularly relevant in the context of MeJA treatment, which is often used to mimic stress responses to induce the production of valuable bioactive compounds. Furthermore, insights gained from studying RNA editing in microalgae could also inform broader applications in other organisms, contributing to advancements in biotechnology and medicine.

Our findings extend beyond metabolite quantification to highlight RNA editing as a key regulatory mechanism underlying MeJA-induced metabolic changes. Our findings reveal that RNA editing significantly impacts the regulation of critical genes associated with fucoxanthin biosynthesis (*Lhcr10*) and lipid metabolism (*PHATR3_J43665*), suggesting that RNA editing is a pivotal mechanism enabling metabolic plasticity under stress conditions.

The light-harvesting complex protein gene *Lhcr10* exhibited significant differential RNA editing in response to MeJA treatment. As a member of the fucoxanthin chlorophyll a/c-binding protein family, *Lhcr10* is directly associated with photosynthetic efficiency and light absorption [51]. RNA editing of *Lhcr10* may enhance its functionality under stress, optimizing the light-harvesting process to support increased fucoxanthin biosynthesis. These findings underline the importance of post-transcriptional modifications in photosynthetic pigment production, providing new insights into the regulation of fucoxanthin biosynthesis in diatoms.

Among the genes linked to lipid metabolism, *PHATR3_J43665* and *PHATR3_J49708* displayed significant RNA editing events. *PHATR3_J43665* exhibited significant differences in RNA editing and expression levels, further implicating its role in lipid metabolism regulation. Functional characterization of this gene may uncover new mechanisms underlying the biosynthesis of fatty acids and lipid storage molecules in diatoms. *PHATR3_J49708* is likely to encode PtDGAT3 (diacylglycerol acyltransferase 3), an enzyme that catalyzes the final step in triacylglycerol biosynthesis [52]. This prediction is supported by its homology to the DGAT3 enzyme [53]. RNA editing in *PHATR3_J49708* could potentially enhance its enzymatic activity or stability, thereby contributing to the observed increase in unsaturated fatty acid production under MeJA treatment.

The identification of RNA editing events in key genes like *Lhcr10* and *PHATR3_J43665* highlights the adaptability of *P. tricornutum* to environmental stressors. RNA editing enables fine-tuning of protein function, complementing transcriptional and translational regulation. This study expands the known roles of RNA editing in diatoms, particularly in optimizing metabolic pathways to balance energy requirements and bioactive compound production under stress.

These findings provide a foundation for exploring RNA editing as a tool for metabolic engineering in diatoms. Targeted manipulation of RNA editing events in *Lhcr10* and lipid metabolism genes like *PHATR3_J43665* and *PHATR3_J49708* could enhance the production of commercially valuable compounds such as fucoxanthin and polyunsaturated fatty acids. For instance, increasing the expression or activity of rate-limiting enzymes through RNA editing could redirect metabolic flux, boosting the biosynthesis of these high-value metabolites while minimizing resource inputs and extraction challenges. This approach addresses the longstanding challenges of low metabolite yield and high extraction costs, presenting RNA editing as a promising biotechnological tool for scalable algal production systems.

Moreover, the impact of RNA editing on lipid metabolism has broader implications for industrial applications, particularly biofuel production. Enhanced lipid biosynthesis is a critical goal for developing cost-effective and sustainable biodiesel. By optimizing key enzymatic pathways, RNA editing could significantly improve lipid accumulation in diatoms, providing a renewable and scalable source of biofuels. Beyond biofuels, the increased production of unsaturated fatty acids offers potential applications in nutraceuticals and pharmaceuticals, broadening the industrial relevance of RNA editing in diatoms.

While this study emphasizes the role of RNA editing, its integration with transcriptomics, proteomics, and metabolomics will provide a more comprehensive understanding of stress responses in diatoms. For example, integrating transcriptomic data with metabolomic profiles could help identify rate-limiting steps in lipid biosynthesis or carotenoid production, enabling targeted interventions to enhance metabolic output. Functional studies of *PHATR3_J43665* and *PHATR3_J49708* could elucidate their precise roles in lipid metabolism, potentially leading to breakthroughs in biofuel production. Furthermore, proteomic analysis could reveal post-translational modifications that synergize with RNA editing to optimize enzyme activity under stress conditions.

One specific example of a breakthrough involves the development of engineered diatoms that exhibit increased triacylglycerol production by simultaneously enhancing the RNA editing levels of *PHATR3_J49708* and overexpressing other rate-limiting enzymes involved in fatty acid elongation. Such integrative approaches could result in diatom strains capable of producing biofuels more efficiently than current models. Similarly, combining RNA editing data with metabolomic insights could allow for the optimization of fucoxanthin biosynthesis by redirecting precursor flux to maximize yield while minimizing cellular energy expenditure.

Elucidating the upstream regulatory mechanisms governing RNA editing under stress conditions will also be critical. For instance, identifying stress-responsive signaling pathways that regulate RNA editing activity could inform the design of robust diatom strains capable of withstanding environmental fluctuations while maintaining high productivity. These approaches could also facilitate the discovery of novel stress tolerance mechanisms, advancing algal biotechnology applications in challenging environmental conditions, such as high salinity or temperature extremes.

In conclusion, this study highlights the critical role of RNA editing in modulating metabolic pathways in *P. tricornutum*. The findings not only deepen our understanding of diatom stress biology but also open new avenues for metabolic engineering and biotechnological exploitation of this versatile microalga.

## 4. Materials and Methods

### 4.1. RNA-Seq Dataset Source

This study utilized raw Fastq sequencing data of *Phaeodactylum tricornutum* provided by our previous study [32]. The sequencing data used for RNA editing analysis contained two experimental groups: a treatment group exposed to 200 μmol/L methyl jasmonate (MeJA) and an untreated control group (*N* = 3 for each group).

### 4.2. Data Pre-Processing

To ensure high-quality data, raw sequencing reads were processed using FastQC (version 0.12.1) for quality assessment, followed by the removal of low-quality reads. High-quality reads were then aligned to the *P. tricornutum* reference genome (JGI Genome Portal: Phycocosm) using HISAT2 (version 2.2.1) [54]. The alignment process generated BAM files, which were subsequently used for downstream analyses. Script development and data pre-processing were performed on a Linux operating system using Python 3 (version 3.8.10).

### 4.3. Identification and Quantification of RNA Editing Events

RNA editing sites were identified using the Genome Analysis Toolkit (GATK) pipeline (version 4.4) with the following filtering criteria: base quality ≥25, sequencing depth ≥ 5, alternative allele depth ≥ 3, and editing frequency between 10% and 100%. High confidence editing events were retained if their editing levels were ≥10% and detected in at least two samples.

The distribution of RNA editing frequencies was visualized for each condition. Principal component analysis (PCA) was conducted on the RNA editing frequency data, with each point in the PCA plot representing one biological replicate. RNA editing events, including stop-gain, synonymous, and non-synonymous mutations, were annotated using Annovar [55].

### 4.4. Differential Editing Analysis and Functional Enrichment Analysis of Differential Editing Events

For each editing site, the number of editing reads was calculated for the MeJA-treated and control groups. At the gene level, total editing reads across all sites within a gene were aggregated to quantify editing activity. Differential RNA editing was analyzed at both the site and gene levels using DESeq2 [56], with adjusted *p*-value < 0.05 considered significant. Site-level analyses identified editing events with significant differences between treatment and control groups, while gene-level analyses highlighted genes with differential editing activities across all sites.

Functional enrichment analysis of differential editing events was performed using the DAVID database, focusing on GO terms and KEGG pathways associated with fucoxanthin biosynthesis and fatty acid metabolism. KEGG pathway analysis primarily used site-level differential editing data due to the limited number of gene-level results.

### 4.5. RNA Expression Quantification and Analysis

Gene expression levels were quantified using StringTie (version 2.2.0) [57] to reconstruct transcripts and calculate TPM values. Differentially expressed genes (DEGs) were identified using DESeq2, with thresholds of FDR < 0.05 and |log_2_FC| > 1.2. Functional enrichment analysis of DEGs was performed using DAVID, focusing on GO terms and KEGG pathways related to fucoxanthin and fatty acid biosynthesis.

### 4.6. Correlation Analysis Between RNA Editing and Gene Expression

An integrative analysis was conducted to explore the relationships between RNA editing and gene expression. Differentially edited sites and DEGs were compared, revealing limited overlap at the gene level compared to the site level.

Quadrant analysis and quadrant classification of differential editing sites and DEGs was performed to identify candidate genes. Correlation analysis was conducted using Pearson correlation. The entire workflow was executed using R (version 4.2.0).

### 4.7. Statistical Analysis

At least three valid repeat tests were performed for each treatment, and the results are expressed as the mean ± SD. Graph analysis and statistical comparisons used GraphPad Prism statistical software, version 9.5.0. The unpaired two-tailed *t*-test (for two groups) and one-way ANOVA (for multiple groups) were used to identify the significance of difference. A probability of less than 0.05 was considered to be statistically significant. * *p* < 0.05, ** *p* < 0.01, *** *p* < 0.001, and **** *p* < 0.00001.

## 5. Conclusions

In summary, this study demonstrates the significant role of RNA editing in regulating stress-induced metabolic shifts in *P. tricornutum*. By identifying specific RNA editing events that enhance fucoxanthin and unsaturated fatty acid synthesis, we provide a novel framework for understanding and leveraging post-transcriptional modifications in algae. These findings open new avenues for optimizing the production of bioactive compounds in marine microalgae, with broad implications for biotechnology and sustainable development.

## Figures and Tables

**Table 1:** RNA editing loci information across samples.

Sample		Control_1	Control_2	Control_3		MeJA_1	MeJA_2	MeJA_3
Editing Loci		87,064	87,151	88,696		74,651	79,182	77,649
	*Total*	262,911				231,482		
Specific Editing Loci	*C to T*	83,450 (31.6%)			*C to T*	73,534 (31.6%)		
	*A to G*	81,934 (31%)			*A to G*	72,790 (31.4%)		

**Table 2:** RNA editing loci information for *Phatr3_J43665*.

Gene	Chrom	*p*-Value (Gene)	Pos	Class	*p*-Value (Loci)	ExonicFunc
*phatr3_j43665*	Chr:2	1.28 × 10^−5^	1138048	G->T	0.019683503	synonymous SNV
			1138360	C->G	0.008246374	nonsynonymous SNV

## Data Availability

Data are contained within the article.

## Supplementary Materials

The following supporting information can be downloaded at: https://www.mdpi.com/article/10.3390/md23020066/s1, Table S1: Detailed RNA editing information for *phatr3_j16481* and *phatr3_j51230* genes in the Lhc gene family; Table S2: Detailed RNA editing information for *phatr3_j49708* gene in the lipid metabolism pathway.
