# Supplementary material for: RNA Editing Analysis Reveals Methyl Jasmonic Acid Regulation of Fucoxanthin and Fatty Acid Metabolism in Phaeodactylum tricornutum
Source: Mar Drugs. 2025 Feb 6;23(2):66. doi: 10.3390/md23020066 (PMC11857586; doi:10.3390/md23020066)
Supplement: Supplementary file 1 [file marinedrugs-23-00066-s001.zip › marinedrugs-3415236-supplementary.pdf]

Table S1 | RNA Editing Loci information across samples.

**Sample**		C1	C2	C3		M1	M2	M3
Editing Loci		87,064	87,151	88,696		74,651	79,182	77,649
	Total		262,911				231,482	
Specific editing loci	*C to T*		83,450 (31.6%)		*C to T*		73,534 (31.6%)	
	A to G		81,934 (31%)		*A to G*		72,790 (31.4%)	

Table S2 | RNA Editing Loci Information for *Phatr3_J43665*

**Gene**	**Chrom**	**P.Value (gene)**	**Pos**	**class**	**P.Value (Loci)**	**ExonicFunc**
Phatr3_J43665	Chr:2	1.28E-05	1138048	G->T	0.019683503	synonymous SNV
			1138360	C->G	0.008246374	nonsynonymous SNV
